# Associations between children’s independent mobility and physical activity

**DOI:** 10.1186/1471-2458-14-91

**Published:** 2014-01-29

**Authors:** Stephanie Schoeppe, Mitch J Duncan, Hannah M Badland, Melody Oliver, Matthew Browne

**Affiliations:** 1 Central Queensland University, School of Human, Health and Social Sciences, Centre for Physical Activity Studies, Building 18, Bruce Highway, Rockhampton QLD 4702, Australia; 2 The University of Melbourne, McCaughey VicHealth Centre for Community Wellbeing, 207 Bouverie Street, Carlton, VIC 3010, Australia; 3 Auckland University of Technology, Human Potential Centre, Mail #A-24, Private Bag 92006, Auckland 1020, New Zealand; 4 Central Queensland University, School of Human, Health and Social Sciences, University Drive, Branyan, Bundaberg QLD 4670, Australia

**Keywords:** Cross-sectional, Active travel, Unsupervised outdoor play, Movement, Young people, Actiheart

## Abstract

**Background:**

Independent mobility describes the freedom of children to travel and play in public spaces without adult supervision. The potential benefits for children are significant such as social interactions with peers, spatial and traffic safety skills and increased physical activity. Yet, the health benefits of independent mobility, particularly on physical activity accumulation, are largely unexplored. This study aimed to investigate associations of children’s independent mobility with light, moderate-to-vigorous, and total physical activity accumulation.

**Methods:**

In 2011 - 2012, 375 Australian children aged 8-13 years (62% girls) were recruited into a cross-sectional study. Children’s independent mobility (i.e. independent travel to school and non-school destinations, independent outdoor play) and socio-demographics were assessed through child and parent surveys. Physical activity intensity was measured objectively through an Actiheart monitor worn on four consecutive days. Associations between independent mobility and physical activity variables were analysed using generalized linear models, accounting for clustered sampling, Actiheart wear time, socio-demographics, and assessing interactions by sex.

**Results:**

Independent travel (walking, cycling, public transport) to school and non-school destinations were not associated with light, moderate-to-vigorous and total physical activity. However, sub-analyses revealed a positive association between independent walking and cycling (excluding public transport) to school and total physical but only in boys (*b* = 36.03, *p* < 0.05). Frequent independent outdoor play (three or more days per week) was positively associated with light and total physical activity (*b* = 29.76, *p* < 0.01 and *b* = 32.43, *p* = 0.03, respectively). No significant associations were found between independent outdoor play and moderate-to-vigorous physical activity. When assessing differences by sex, the observed significant associations of independent outdoor play with light and total physical activity remained in girls but not in boys. All other associations showed no significant differences by sex.

**Conclusions:**

Independent outdoor play may boost children’s daily physical activity levels, predominantly at light intensity. Hence, facilitating independent outdoor play could be a viable intervention strategy to enhance physical activity in children, particularly in girls. Associations between independent travel and physical activity are inconsistent overall and require further investigation.

## Background

In Australia, 60% of children aged 9-13 years do not meet current national guidelines for physical activity recommending that children engage in at least 60 minutes of moderate-to-vigorous physical activity every day [1]. Habitual physical activities such as playing outdoors, and walking and cycling for transport provide many opportunities to be physically active throughout the day [2]. Physical activity is vital for children’s bone health, motor skills, physical fitness, healthy weight and protection against chronic diseases later in life [3-7]. Instilling habitual physical activity in young children is crucial as physical activity behaviours tend to track from childhood to adolescence and adulthood [8].

Children’s independent mobility describes the freedom of those aged under 18 years to play outdoor and travel to places without adult supervision [9,10]. Independent mobility provides an important source for children’s habitual physical activity. Children’s unsupervised neighbourhood play and exploration brings psychosocial, cognitive and developmental benefits in the form of social interactions with peers, spatial and traffic safety skills for navigating in public spaces, and maturity in regard to decision making [11-13]. Independent mobility starts to increase between the ages 8-13 years, often coinciding with the transition from primary to secondary school; this is in response to parents recognising increasing physical and cognitive capabilities as children age [10,14]. Boys tend to have higher levels of independent mobility compared to girls [15-18]. This can be explained by parent’s tendency to be more protective towards girls than boys, and differences in socialising between boys and girls [18]. For example, boys tend to form casual friendships through local outdoor activities such as football which requires independent mobility nearby the home [18]. In contrast, girls tend to form close friendships through talking and socialising at friend’s home, shopping centres or cinemas which often requires motorised transport [18]. For children, independent travel usually involves active modes of travel such as walking and cycling, as well as using public transport [19]. In many developed countries including the United States of America, Australia, Italy, Denmark, Finland and Norway, children’s walking and cycling for transport has dramatically declined [14,19-22]. Long-term trend data from England showed that in 1971, 86% of primary school children were allowed to travel home from school alone, whereas in 1990 this proportion was 35%, and in 2010 it was only 25% [23]. Reasons for the decreases in children’s independent mobility include parental concerns about road safety and stranger danger, greater complexity in families’ daily schedules, and longer travel distances to schools, shops and recreational facilities [14,15,24,25]. The declines in independent mobility are concerning from a child development and public health perspective including contributions to low levels of physical activity in children.

Few studies have investigated the potential of independent mobility to enhance children’s physical activity levels [26]. Three studies focused on associations between parental licences for their child’s independent travel (but not actual travel behaviour) in public spaces and physical activity, finding significant positive associations [15,16,27]. Another study [28] examined children’s independent outdoor play in parks, playgrounds and other open spaces; findings showed that children were more physically active when playing solely in the presence of peers compared with adults. However, the small number of existing studies in this research field limits the generalisability of findings. Moreover, existing studies have not differentiated between independent mobility domains (travel, outdoor play) and physical activity intensities (light, moderate-to-vigorous) [15,16,27]. Evidence on whether independent mobility enhances physical activity across various intensities is valuable since both light and moderate-to-vigorous physical activity provide health benefits in children [5,7,29]. Notably, light physical activity has been ignored in previous studies though it is associated with improvements in blood pressure, insulin and HDL-cholesterol levels in children; health benefits which are particularly important for overweight and obese children in whom metabolic risk factors are increasingly prevalent [30,31].

This paper aims to extend the current evidence base by

(1) investigating associations between children’s self-reported independent mobility (including independent travel and outdoor play) and objectively measured light, moderate-to-vigorous, and total physical activity,

(2) testing whether associations between children’s independent travel and physical activity would differ when independent travel involves walking and cycling versus walking, cycling and public transport,

(3) examining differences in all observed associations by sex.

As such, information gathered from this study could help design interventions to promote children’s independent mobility with the aim to enhance habitual physical activity at various intensities.

## Methods

### Study design

This cross-sectional study is based on data drawn from the Australian CATCH/iMATCH projects (Children's Activity, Travel, Connectedness and Health/Independent Mobility, Active Travel and Children’s Health). The interrelated CATCH/iMATCH projects were designed to investigate the role of policy, social, and built environments in influencing children's independent mobility, active travel, and related health outcomes [32]. Ethical clearance for conducting the CATCH/iMATCH projects was obtained from several universities (Central Queensland University, H10/11-170; The University of Melbourne, HREC 1135410; Curtin University, HR 140/2010; Griffith University ENV/31/10/HREC) and State Departments of Education and Training in Australia (Queensland (QLD), 550/27/1042; Victoria (VIC), 2011-001232; Western Australia (WA), D11/0698973).

### Participants

A clustered sampling design and convenience sample were used in the CATCH/iMATCH projects. Overall, 375 children aged 8-13 years were recruited from nine public primary schools in Rockhampton (QLD), Brisbane (QLD), Melbourne (VIC) and Perth (WA). The schools were located in neighbourhoods with various levels of urbanisation (inner urban, n = 2; middle suburban, n = 2; outer suburban, n = 2; regional area, n = 3) but similar socio-economic status (SES) characteristics of student populations (representing a middle SES range). The selection of schools was guided by SES information available from Australia’s My School website (http://www.myschool.edu.au) which determined SES via several socio-demographic factors such as parents’ occupational level and level of educational attainment. School principals, teachers, parents and students (Years 3-7) were briefed about the study through informational materials and meetings held at the schools. Written consent by school principals, parents and children was a requirement for study participation.

### Data collection

Data collection took place between June 2011 and November 2012 including the Australian winter and summer. Under researchers’ supervision, students completed a paper-based survey at school, either in class or during lunch recess. The paper-based survey administered to children assessed socio-demographics, travel and independent mobility behaviours. A paper-based survey administered to parents included information on socioeconomic status. Furthermore, children wore an Actiheart, a combined heart rate and accelerometer monitor (CamNtech Actiheart, Cambridge, UK), for four consecutive days, except during periods of heavy contact sports and water-based activities (e.g. showering, swimming). The Actiheart monitoring period included weekdays and weekend days, either consecutively from Thursday until Monday morning or from Friday until Tuesday morning. The Actiheart was attached directly onto the skin on the lower left side of the chest using two electrocardiograph (ECG) electrodes (model Red Dot 2238 soft cloth, 3 M) [33,34]. One electrode was placed at the base of the child’s sternum and the other electrode placed horizontally to the left so the wire of the Actiheart was straight with tension, but not tight. Researchers demonstrated to the students how the Actiheart should be worn. The students then fitted the units and researchers checked the positioning.

#### Socio-demographic assessment

Sex and age in years was captured through the child surveys. Additional covariates were assessed through parent surveys administered via mail. These included parental education attainment and number of motor vehicles in the household. In this study, neighbourhood urbanisation in state capital city locations (Brisbane, Melbourne, Perth) was defined based on neighbourhood proximity to the central business district (CBD) with the general post office being a marker of the CBD centre. Inner urban, middle suburban, and outer suburban were defined as being within 5 km from CBD, 11-20 km from CBD and beyond 20 km from CBD, respectively. Regional area was defined as a regional town that is not a major state capital city of Australia.

#### Independent mobility assessment

Independent mobility was assessed through child self-report surveys; complementary parental proxy reports of children’s independent mobility behaviour could not be collected due to space restrictions in parent surveys. In the absence of standardised, validated measures of independent mobility, questions were derived from child surveys used in similar studies [9,35] to capture (a) travel mode and accompaniment of active travel, and (b) frequency and accompaniment of outdoor play (predictor variables).

Children were asked ‘How do you usually travel to: (1) school; (2) local shops; (3) local friend’s houses; (4) local parks and playgrounds; and (5) organised activities (e.g. at a local sports club, church or recreational centre)?’ Children selected the response option best representing their predominant travel mode (walk, bicycle, take public transport, be driven) and accompaniment status (alone, with other children, with an adult) for each destination. Questions relating to non-school destinations also included the option ‘Don’t go there’. Responses to these five destinations were collapsed to create two variables: (1) usual travel to school; and (2) usual travel to local non-school destinations. Response options for the ‘usual travel to school’ variable were dichotomised into ‘independently mobile’ (i.e. walk/bicycle/take public transport alone or with other children) or ‘not independently mobile’ (i.e. walk/bicycle/take public transport or car with an adult). Using this same classification of independent mobility the ‘usual travel to local non-school destinations’ variable was calculated as the ratio of independently mobile trips from all trips a child undertook to non-school destinations. Based on the median distribution this variable was then dichotomised into ‘higher levels of independent travel’ (i.e. 33-100% of all trips occur independently mobile) or ‘lower levels of independent travel’ (i.e. < 33% of all trips occur independently mobile).

Children were also asked ‘How often do you usually play outdoors in your neighbourhood, for example, on the street, in a nearby park, bush area, or on a playground?’ Children selected from the following response options: ‘5 or more days per week’; ‘3-4 days per week’; ‘1-2 days per week’ and ‘Never’. If children played outdoors they also reported on their usual accompaniment status: alone; without an adult present but with other children; or always with an adult present irrespective of other children. In this sample, all children responded that they play outdoors independently (i.e. alone or with other children). Based on the median distribution the response categories were dichotomised into ‘higher levels of independent outdoor play’ (i.e. played ≥ 3 days per week, alone or with other children) or ‘lower levels of independent outdoor play’ (i.e. never or played 1-2 days per week, alone or with other children).

#### Physical activity assessment

Light, moderate, vigorous, and total physical activity (outcome variables) were assessed using accelerometer counts derived from the Actiheart unit. The Actiheart accelerometer has demonstrated good validity (R^2^ = 0.7) when tested with 13-year old children in a laboratory setting [36]. At the conclusion of wearing the Actiheart units, data were downloaded using the CamNtech Actiheart software. Subsequently, the Actiheart data was transferred into Microsoft Excel and data reduction was carried out to extract daily minutes spent in light, moderate and vigorous physical activity out using custom written software (National Instruments Labview). *Wear time and non-wear time:* Actiheart recordings were classified by the Actiheart software as being ‘OK’, ‘recovered’, ‘interpolated’, ‘lost’ or ‘not worn’. Only accelerometer counts recorded under the classifications ‘OK’, ‘recovered’ or ‘interpolated’ were used for calculating time spent in physical activity; this shows that the Actiheart was detecting a signal and suggests wear time. Actiheart recorded Recordings under the classifications ‘lost’ and ‘not worn’ suggest non-wear time or the units were not detecting a signal; hence these were excluded from analyses. *Epoch length:* The epoch length was aggregated to 60 seconds to account for differences in epoch length settings across sites. Sixty seconds epochs have shown to be appropriate for measuring moderate-to-vigorous physical activity in children [37] and allowed comparison with similar studies [38-41]. *Cut-points:* For inclusion in analyses, children were required to record accelerometer counts on at least two days for a minimum of 360 minutes per day between the hours of 6.00 am-11.00 pm. This threshold combination was used based on availability of valid Actiheart accelerometer data and sensitivity analyses. The child-specific ActiGraph cut points developed by Evenson et al. [42] were adopted for classifying light, moderate and vigorous physical activity (being 101-2295, 2296-4011 and ≥ 4012 counts per minute, respectively). These values showed acceptable classification accuracy for light, moderate and vigorous physical activity with ROC-AUC (area under the receiver operating characteristic curve) curve values ranging between 0.70-0.84 [42,43]. Following the protocols of Ridgway et al. [44] a conversion factor of five (i.e. ActiGraph counts = Actiheart counts x 5) was applied to generate comparable cut points for Actiheart data, resulting in final cut points for light, moderate and vigorous physical activity of 21-459, 460-802 and ≥ 803 counts per minute, respectively. Average number of minutes per day spent in light, moderate-to-vigorous (i.e. moderate and vigorous physical activity combined) and total physical activity were extracted.

### Statistical analyses

Randomly missing data on independent travel to school and non-school destinations, independent outdoor play and socio-demographics were handled using multiple imputation following Rubin’s [45] conventional method. This involved three steps: (1) creating five data sets with imputed values for the relevant independent mobility and socio-demographic variables with consideration of all variables to be included in analyses; (2) conducting the analyses in all data sets; (3) calculating averages from each data output. Missing values were imputed via 5-fold multiple imputation involving repeated sampling the empirical multivariate distribution using the Amelia package [46] within R statistical programming environment [47]. Reported coefficient means and standard-errors were aggregated across imputed data-sets. Chi-square tests and independent t-tests were conducted to assess differences in socio-demographics, independent mobility and time spent in physical activity (moderate-to-vigorous physical activity was log-transformed) between boys and girls, and between children included and excluded from analyses. Subsequently, a series of regression analyses were carried out using generalized linear models to examine associations between children’s independent mobility and time spent in light, moderate-to-vigorous and total physical activity. Analyses were adjusted for clustering by school location using robust standard errors; the school location represented neighbourhoods with various levels of urbanisation. Included covariates were age, parental education attainment, number of motor vehicles in the household and Actiheart wear time. Given documented sex differences in children’s independent mobility and physical activity [16,18] an interaction term was entered in the model to test for differences by sex. Furthermore, all analyses were repeated in a sub-sample of children (n = 179) that excluded those who travelled by public transport (n = 12). These sub-analyses tested whether associations between independent travel and physical activity differ when focusing specifically on independent travel by walking and cycling. Associations are presented using unstandardised beta coefficients (b), standard errors, confidence intervals and p-values. The significance level was set at 0.05. Analyses were performed in IBM SPSS Statistics (version 19.0) and StataMP (version 12.0).

## Results

### Study population

Of approximately 1,534 eligible children invited across all participating schools, 375 (24%) children provided written parental consent and child assent to study participation. Thirteen children dropped out of the study because they either left the school or withdrew from data collection. Actiheart data were non-valid (i.e. < 2 days of accelerometer recordings for at least 360 minutes between 6.00 am-11.00 pm) in 136 children, and imputation of independent mobility and socio-demographic data was not suitable for 35 children because responses such as ‘Other’ and ‘Don’t go there’ could not be classified into available response categories. Hence, these children were excluded from analyses. After data imputation in 65 cases, the final sample included in analyses was 191 (51%) out of 375 recruited children.

There were no significant differences in socio-demographics, levels of independent mobility, or physical activity between children included and excluded in analyses (data not reported), except for neighbourhood urbanisation (*p* < 0.01). A higher proportion of excluded children were from schools in outer suburban neighbourhoods (64.9% versus 35.1%), and a lower proportion were from schools in inner urban (35.7% versus 64.3%) and regional neighbourhoods (27.6% versus 72.4%). Table 1 shows descriptive characteristics of the study participants included in analyses. Overall, 36% of children commuted independently to school and 43% usually travelled independently to non-school destinations. Sub-analyses of travel modes showed that most children (57%) were driven to school and non-school destinations (data not presented in Table 1). All children engaged in independent outdoor play; 67% of them at higher levels (≥ 3 days per week). There were no significant differences in socio-demographics, independent mobility and physical activity levels between boys and girls.

**Table 1:** Descriptive characteristics of the study participants included in analyses

	**All**	**Boys**	**Girls**	***p*-value**
**N (%)**	191	73 (38.0)	118 (62.0)	
**Age in years, mean (SD)**	10.64 (0.89)	10.76 (0.91)	10.57 (0.87)	0.15
**Level of urbanisation, n (%)**				
Inner urban	54 (28.3)	22 (30.1)	32 (27.1)	
Middle suburban	35 (18.3)	14 (19.2)	21 (17.8)	
Outer suburban	47 (24.6)	14 (19.2)	33 (28.0)	
Regional area	55 (28.8)	23 (31.5)	32 (27.1)	
**Parental education attainment**^**1**^**, n (%)**				0.48
High school	63 (32.9)	25 (34.2)	38 (32.2)	
Trade/apprenticeship/certificate/diploma	72 (37.9)	24 (32.9)	48 (40.7)	
University degree	56 (29.2)	24 (32.9)	32 (27.1)	
**Motor vehicles in household, n (%)**				0.79
0	3 (1.6)	1 (1.4)	2 (1.7)	
1	45 (23.6)	20 (27.4)	25 (21.2)	
2	109 (57.0)	40 (54.8)	69 (58.5)	
≥ 3	34 (17.8)	12 (16.4)	22 (18.6)	
**Usual travel to school, n (%)**				0.88
IM	69 (36.1)	27 (37.0)	42 (35.6)	
Non-IM	122 (63.9)	46 (63.0)	76 (64.4)	
**Usual travel to non-school destinations, n (%)**				0.47
Higher IM	82 (42.9)	35 (47.9)	47 (39.8)	
Lower IM	109 (57.1)	38 (52.1)	71 (60.2)	
**Usual outdoor play, n (%)**				0.77
Higher IM	126 (66.5)	46 (63.0)	80 (67.8)	
Lower IM	65 (33.5)	27 (37.0)	38 (32.2)	
**Average daily minutes in light PA**				
Mean (SD)	381.57 (95.72)	375.87 (86.7)	384.67 (89.90)	0.55
**Average daily minutes in MVPA**				
Mean (SD)	57.22 (45.90)	64.60 (48.59)	52.65 (43.80)	0.08
Median (IQR)^2^	45.30 (44.80)	52.00 (50.30)	42.40 (40.30)	0.07
**Average daily minutes in total PA**				
Mean (SD)	438.90 (95.3)	440.47 (110.0)	437.32 (118.50)	0.85

*Abbreviations:IM* independent mobility, *IQR* interquartile range, *MVPA* moderate-to-vigorous physical activity, *PA* physical activity, *SD* standard deviation.

^1^Highest education attainment was reported by one parent completing a survey.

^2^The distribution of MVPA was positively skewed; hence means, SD, medians and IQR are presented. MVPA was log-transformed prior to conducting independent *t*-test.

### Associations between independent mobility and physical activity

Table 2 presents associations between independent mobility and daily time spent in light, moderate-to-vigorous, and total physical activity. Compared to adult-accompanied travel, children’s independent travel (walking, cycling, public transport) to school and non-school destinations were not significantly associated with light, moderate-to-vigorous and total physical activity. However, children who frequently engaged in independent outdoor play (≥ 3 days per week) accumulated more daily minutes of light physical activity (*b* = 29.76, *p* < 0.01) and total physical activity (*b* = 32.43, *p* = 0.03) than those who played outdoors less often. The frequency of independent outdoor play was not significantly associated with moderate-to-vigorous physical activity.

**Table 2:** **Associations between independent mobility and mean daily minutes spent in light, moderate-to-vigorous and total physical activity**^**1**^

	**Light PA**				**MVPA**				**Total PA**			
	***b***	**SE**^**2**^	**95% CI**	***p*-value**	***b***	**SE**	**95% CI**	***p*-value**	***b***	**SE**	**95% CI**	***p*-value**
**Usual travel to school**												
IM (n = 68)	-0.55	9.05	-19.12 – 18.02	0.95	-1.48	5.11	-12.86 – 9.90	0.80	-2.53	11.35	-25.92 – 20.85	0.83
Non-IM (n = 123)	–		–	–	–		–	–	–		–	–
**Usual travel to non-school destinations**												
Higher IM (n = 82)	-13.24	8.95	-35.85 – 9.37	0.25	4.81	7.76	-11.17 – 20.78	0.56	-4.10	15.44	-39.96 – 31.76	0.82
Lower IM (n = 109)	–		–	–	–		–	–	–		–	–
**Usual outdoor play**												
Higher IM (n = 127)	29.73	9.57	9.39 – 50.06	<0.01	3.96	7.75	-12.01 – 19.93	0.63	32.43	13.53	3.23 – 61.62	0.03
Lower IM (n = 64)	–		–	–	–		–	–	–		–	–

*Abbreviations:b* unstandardised beta coefficient, *95% CI* 95% confidence interval, *p p*-value, *SE* robust standard error, *IM* independent mobility, *MVPA* moderate-to-vigorous physical activity, *PA* physical activity.

^1^Adjusted for clustering by school location (representing levels of neighbourhood urbanisation), age, sex, parental education attainment, number of motor vehicles in the household, Actiheart wear time.

^2^Standard errors, confidence intervals and *p*-values were calculated from imputed beta coefficients using Rubin’s [38] conventional method.

Note, independent travel to school and non-school destinations included walking, cycling and public transport.

We tested whether the associations between independent travel and physical activity would differ when excluding travel involving public transport. Findings differed only slightly; independent outdoor play remained significantly associated with light physical activity (*b* = 26.62, *p* < 0.05) but not with total physical activity (*b* = 28.26, *p* = 0.13). All other associations remained non-significant.

### Differences by sex

Analyses by sex showed no significant associations of independent travel (walking, cycling, public transport) to school and non-school destinations with light, moderate-to-vigorous and total physical activity. The observed positive associations between frequent independent outdoor play (≥ 3 days per week) and light and total physical activity were significant in girls (*b* = 41.41, *p* < 0.01 and *b* = 46.02, *p* = 0.01, respectively) but not in boys.

We tested whether the associations between independent travel and physical activity would differ when excluding travel by public transport. Results differed slightly. Independent walking and cycling to school was positively associated with total physical activity but only in boys (*b* = 36.03, *p* < 0.05), not in girls. Positive associations of frequent independent outdoor play (≥ 3 days per week) with light and total physical activity were significant in girls only (*b* = 48.46, *p* < 0.001 and *b* = 41.95, *p* = 0.03, respectively). All other associations were non-significant.

## Discussion

This study investigated associations between children’s independent mobility and light, moderate-to-vigorous, and total physical activity. In this sample, children’s independent travel (walking, cycling, using public transport) to school and non-school destinations was not related to higher levels of physical activity compared to adult-accompanied travel. These findings were unexpected and contrary to those found in similar studies [15,16,27]. There are several possible explanations for these findings. Firstly, previous studies did not examine physical activity intensities (light, moderate-to-vigorous) as an outcome but overall physical activity or physical activity types (walking, outdoor play). Secondly, the leisure-time activities children pursue at non-school destinations will likely also influence their physical activity levels [48], besides the commuting itself. However, detailed information on what leisure-time activities children specifically engaged in at non-school destinations was not available for analysis in this study but is worth examining in future research. Thirdly, associations between independent travel and physical activity can differ when focusing specifically on children’s independent walking and cycling for transport. For example, this study revealed a significant positive association between independent walking and cycling to school and total physical activity; however, only in boys. This suggests that significant increases in physical activity generated by independent travel require active modes of travel. Moreover, boys rather than girls may increase physical activity levels from independent travel, possibly because parents tend to grant boys more independence for travels in local areas whereas parents tend to be more restrictive towards girls [18].

Children who frequently engaged in independent outdoor play (≥ 3 days per week) accumulated significantly more minutes of light and total physical activity than those who engaged in it less frequently. In contrast, no significant associations were observed between children’s frequency of independent outdoor play and moderate-to-vigorous physical activity. The difference in associations across physical activity intensities may be explained by built-environmental and social-environmental factors. For example, wide open spaces such as green areas, parks, playgrounds and quiet streets have eroded over time, and public play areas have become less accessible on foot for children due to increased traffic volume and car-oriented urban planning [49]. Additionally, parental concerns about road safety coupled with fears about stranger danger have led to many parents restricting children’s freedom to play in open spaces beyond the close confines of the home garden and yards [49]. These various environment impediments for accessing outdoor areas outside of the home may reduce children’s engagement in outdoor play at higher intensities [50].

Detailed analyses by sex showed that the observed positive associations between independent outdoor play and light and total physical activity remained significant in girls but not in boys. This suggests that independent outdoor play is particularly important for physical activity accumulation in girls. A reason may be that parents’ tend to be more protective towards girls than boys; hence, restrictions of independent mobility usually affect girls more than boys [18,51]. As such, for girls, independent outdoor play in the home garden and yards might provide an important source for physical activity engagement, and an opportunity for talking and socialising with friends [51]. This may be less relevant for boys who, compared to girls, accumulate physical activity more often through organised sports, particularly around the ages 9-11 [51,52].

### Strengths, limitations and future research

Strengths of this study include objective physical activity measurement across various physical activity intensities and examination of diverse children’s independent mobility behaviours (outdoor play, travel to school and non-school destinations), rather than independent mobility licences that have been previously examined [15,16,27]. The distinction between ‘licences’ and ‘actual behaviours’ is important and requires more attention in future studies as children may be allowed to travel independently to some places but end up being driven because it is faster, more convenient and more comfortable than walking or cycling. Other methodological strengths include the use of multiple imputation techniques to account for randomly missing data, sensitivity analyses to help determine thresholds for valid Actiheart data, adjustment for a range of potential confounders, and testing for differences by independent travel modes and sex. This study also had several limitations. Social and environmental determinants such as perceptions of neighborhood safety, distances to travel destinations, weather, street connectivity, availability of cycling and walking trails were not considered in this study though these will likely influence children’s levels of independent mobility and physical activity [52,53]. Our analyses accounted for neighbourhood urbanisation though. Other limitations of this study include the cross-sectional study design, the reliance on non-standardised children’s self-report measures for assessing independent mobility, the low response rate and low adherence to wearing an Actiheart monitor. The latter resulted in a short physical activity monitoring period which may not have fully recorded children’s usual physical activity patterns. Moreover, the use of a small convenience sample limits the generalisability of findings. Although a 60 seconds epoch length has shown to be appropriate for measuring moderate-to-vigorous physical activity in children [37], a lower epoch length may have better captured children’s intermittent and spontaneous physical activity patterns [54]. We could not use a shorter epoch length as epoch lengths differed across the study sites. Overall, we suggest that associations between children’s independent mobility and physical activity receive further attention in future research given the potential of independent mobility to enhance habitual physical activity. This may be particularly important for children who do not obtain physical activity through organised sports. The low response rate found in this study demonstrates the common challenge of recruiting children into behavioural risk factor studies; however, little guidance exists on recruitment methods [55]. More evidence should be gathered on successful recruitment strategies applied in pediatric populations [56]. Furthermore, the application of standardised multiple imputation techniques rather than simple complete cases analysis is advisable in studies with large amounts of missing data [57]. Imputation of missing accelerometer data is emerging but not yet standardised [58], hence it was not carried out in this study.

## Conclusions

In this study, children’s independent travel in the form of walking, cycling and public transport was not correlated with significant gains in daily physical activity. However, independent travel including solely walking and cycling showed a positive association with total daily physical activity but only in boys. Frequent independent outdoor play was significantly associated with accumulation of light intensity physical activity. Hence, promoting independent outdoor play can be a viable intervention strategy to enhance physical activity in children, especially in girls. Further investigations of independent travel by transport mode, the distances away from home children are allowed to travel and play outdoors independently, and the physical and social environments that support these behaviours are needed to inform future interventions.

## Abbreviations

HDL-cholesterol: High-density lipoprotein cholesterol; CATCH project: Children's activity, travel, connectedness and health project; iMATCH project: Independent mobility, active travel and children’s health project; QLD: Queensland; VIC: Victoria; WA: Western Australia, SES, Socio-economic status; ECG: Electrocardiograph; ROC: Receiver operating curve; PA: Physical activity; MVPA: Moderate-to-vigorous physical activity; IM: Independent mobility; SD: Standard deviation; IQR: Interquartile range; b: unstandardised beta coefficient; SE: robust standard error; 95% CI: 95% confidence interval; p: *p*-value.

## Competing interests

The authors declare that they have no competing interests.

## Authors' contributions

SS, MD, HB and MO participated in the conception of the research questions, measurements and interpretation of data. SS contributed substantially to participant recruitment and data collection, and carried out data analyses, with substantive input to methodology and analysis from MD, HB, MO and MB. MB conducted the multiple imputation procedure and provided statistical advice for analyses. SS coordinated the drafting of the manuscript and obtained contributions from all authors. All authors read and approved the final manuscript.

## Pre-publication history

The pre-publication history for this paper can be accessed here:

http://www.biomedcentral.com/1471-2458/14/91/prepub
